# The Effects of *Semen Ziziphi Spinosae* Extract on LPS-Induced Astrocyte Gene Expression and Metabolites

**DOI:** 10.3390/nu17223498

**Published:** 2025-11-07

**Authors:** Jingxuan Ma, Ru Wang, Yaping Xu, Yan Wang, Zixuan Liu, Zhaoxia Wu, Yuanyuan Bian

**Affiliations:** 1 College of Food Science, Shenyang Agricultural University, Shenyang 110866, China; mjx0615@stu.syau.edu.cn (J.M.); 13555720381@163.com (R.W.); wangyan_0913@163.com (Y.W.); leo98729@163.com (Z.L.); 2 Department of Agricultural, Forestry, and Economic, Chaoyang Engineering Technical School, Chaoyang 122000, China; xyp0110@163.com

**Keywords:** astrocytes, *Semen Ziziphi Spinosae*, energy metabolism, ABC transporters

## Abstract

**Background**/**Objectives**: *Semen Ziziphi Spinosae* (SZS), a medicinal and edible traditional Chinese herb, has been widely used to treat insomnia. As critical regulators of the central nervous system, astrocytes play a pivotal role in maintaining sleep homeostasis. However, the mechanisms by which SZS modulates astrocytic function to improve sleep remain unclear. **Methods**: In this study, we employed an integrated transcriptomics and metabolomics approach to investigate the protective effects of SZS extract against lipopolysaccharide (LPS)-induced inflammatory injury and metabolic dysfunction in astrocytes. **Results**: Transcriptomic analysis revealed that SZS ameliorates cellular damage (including apoptosis, autophagy, and cell cycle dysregulation) through a FOXO3-centric signaling network. Concurrently, SZS restored cellular energy metabolism by increasing ATP production and reducing Ca^2+^ overload, thereby activating the AMPK signaling pathway to support normal astrocytic function. Metabolomic profiling further demonstrated that SZS-mediated restoration of energy homeostasis sustains ABC transporter activity, which in turn modulates neurotransmitter (serotonin, L-glutamic acid, adenosine), metabolic mediators (leukotrienes, palmitoylethanolamide, succinic acid), and nucleotide (uridine 5′-diphosphate). These coordinated changes normalized GABAergic synapse activity and neuroactive ligand receptor interactions, ultimately resolving neural metabolic network disturbances. **Conclusions**: Our findings elucidate a novel FOXO3-energy metabolism-ABC transporter axis through which SZS extract attenuates neuroinflammation and metabolic dysfunction in astrocytes and exerts sleep-promoting and neuroprotective effects. This study provides a scientific foundation for understanding the modern pharmacological mechanisms of traditional Chinese medicine in insomnia treatment, highlighting astrocytic regulation as a potential therapeutic target.

## 1. Introduction

Insomnia, as one of the most prevalent neurological disorders, affects approximately 10% of the global population [1]. This disorder is a complex physiological phenomenon resulting from multifactorial interactions, primarily influenced by psychological, physiological, and environmental factors, and is also associated with neuroinflammation and astrocytic dysfunction. Neuroinflammation, an immune response of the nervous system to injury or infection, is characterized by neuronal damage and glial cell activation, leading to the release of pro-inflammatory cytokines (e.g., IL-1β, TNF-α, IL-6) and subsequent disruption of the neuronal microenvironment [2]. Exogenous pro-inflammatory compounds, such as lipopolysaccharide (LPS) [3], can trigger such inflammatory responses. Through blood–brain barrier penetration or neuro-immune interactions, these compounds alter neuronal firing rates in sleep- and wakefulness-related brain structures, including the basal forebrain and hypothalamus in rats [4], thereby inducing sleep fragmentation, difficulty falling asleep, and other sleep disturbances [5].

As the most abundant glial cells in the central nervous system, astrocytes not only regulate the sleep–wake cycle but also participate in neuronal metabolism and synaptic plasticity [6]. Experimental and clinical studies suggest that insomnia, sleep deprivation, and circadian rhythm sleep alterations may directly or indirectly exacerbate neuroinflammation through astrocyte activation, promoting a chronic inflammatory state that negatively impacts neuroplasticity and contributes to neuronal damage [7]. Recent findings further support this mechanism: sleep fragmentation is significantly correlated with upregulated expression of astrocyte activation-related genes in human brain (IFI16, TPST1, CCL2, ZFP36, OSMR, SLC44A3, ANO6, FAM189A2, C4orf19, RANBB9, GGPS1, UST, and NRP1) [8], and the aberrant expression of these genes may reflect impaired astrocytic function [9]. Therefore, effectively preventing and mitigating astrocytic damage caused by neuroinflammatory environments holds significant scientific and potential clinical value.

In recent years, there has been growing interest in the neuroprotective effects of traditional medicines and natural bioactive compounds [10,11], as they exhibit fewer side effects and better biocompatibility compared to synthetic drugs in treating psychiatric disorders such as insomnia, anxiety, and depression [12,13]. *Ziziphus jujuba* Mill. var. *spinosa* (Bunge) Hu ex H.F. Chou, known as *Semen Ziziphi Spinosae* (SZS), is a plant with a long history of use as both food and medicine in China. It was initially recorded in the publication *Shennong Bencao Jing* and possesses significant application and research value [14]. Its sedative, anxiolytic, neuroprotective, and hypnotic effects have been extensively validated [15]. As a functional food, SZS is rich in bioactive compounds, including saponins, flavonoids, and alkaloids, which contribute to its ability to protect cellular function and repair damage. For instance, a previous study has shown that SZS can enhance cell viability, suppress apoptosis, and activate the AMPK/SIRT1/PGC-1α signaling pathway in an in vitro H9c2 rat cardiomyocyte model of ischemia-hypoxia injury [16]. Additionally, jujuboside A, a major active component of SZS, inhibits apoptosis in renal tubular epithelial cells of diabetic mice and ameliorates mitochondrial dysfunction in both mouse renal tubular epithelial cells and high-glucose-cultured HK-2 cells [17]. Although in vitro studies on the functional activities of SZS have been reported, research on its protective effects against glial cell injury remains limited. Our previous study confirmed that the 50% ethanol extract of SZS demonstrates the multi-component synergistic effects of traditional Chinese medicine, which can effectively alleviate LPS-induced astrocyte damage and functional alterations, although the mechanisms behind this effect are not yet fully understood.

Therefore, in this study, we systematically characterized the gene expression profiles and metabolic patterns in LPS-injured astrocytes treated with 50% ethanol extract of SZS. Our comprehensive analysis revealed the responsive mechanisms through which SZS extracts ameliorate LPS-induced astrocytic damage, providing novel scientific evidence for their neuroprotective effects and identifying potential therapeutic targets for treating related neurological disorders.

## 2. Materials and Methods

### 2.1. Preparation of SZS Extract

SZS was purchased from Fengliao Xing (Foshan, China) Pharmaceutical Co., Ltd. In brief, 100 g of SZS was pulverized and sieved through a 60-mesh screen and after defatting with petroleum ether, added 10 times the volume of 50% ethanol to the material. Three successive reflux extractions were performed, each lasting 1 h. The filtrates were combined and concentrated using a rotary evaporator until completely solvent free, resulting in a 50% ethanol extract with a yield of 18.9%, which was designated as S50. In our previous laboratory studies, it was confirmed by HPLC that S50 contains jujuboside A and spinosin at concentrations of 4.40 mg/g and 9.76 mg/g, respectively. Additionally, the total saponin content of S50 was determined to be 71.92 mg/g (expressed as ginsenoside Rb1 equivalents), the total flavonoid content of S50 was determined to be 13.26 mg/g (expressed as rutin equivalents).

### 2.2. Cell Culture and Treatments

The rat brain type I astrocyte cell line (CTN-TNA2) derived from ATCC was obtained from Yuchi Biotechnology Co., Ltd. (Shanghai, China; catalog number SC1460). Astrocytes were cultured in DMEM medium supplemented with 9% fetal bovine serum (FBS) and 1% penicillin–streptomycin at 37 °C under 5% CO_2_ and 95% air. For subsequent experiments, cells in the logarithmic growth phase were inoculated according to the required amount and cultured for 24 h. After that, the culture medium was changed to serum-free DMEM solution and starved for 24 h. Then, the cells were divided into three groups: (1) CK group, (2) LPS group, and (3) LPS + S50 (L50) group. The CK group was cultured in a complete medium, while the LPS group was treated with 1 μg/mL LPS solution (complete medium preparation) to induce an inflammatory model. The L50 group was pretreated with 1 mg/mL S50 solution (the concentration was selected on the basis of our preliminary cell viability studies (Supplementary File S1: Supplementary Methods and Table S1) and literature review [18]) for 1 h before LPS induction. All groups underwent subsequent incubation for 24 h under the aforementioned culture conditions.

### 2.3. Transcriptomics Analysis

Following cell counting, cells were seeded at a density of 1 × 10^6^ cells/well in 6-well plates. After adhesion, cells were subjected to experimental treatments before harvest. Cell integrity was verified via microscopy. Rinse adherent cells twice with ice-cold 1× PBS (prepared with DEPC-treated water). Add 1 mL TRIzol™ reagent (Invitrogen, Carlsbad, CA, USA) to each well. Homogenize cells through repetitive pipetting until complete lysis is achieved (visually confirmed by clear, non-viscous solution formation). Transfer lysates to RNase-free microcentrifuge tubes. Flash-freeze in liquid nitrogen and store at −80 °C for subsequent RNA detected.

The concentration and purity of RNA were determined using a NanoDrop 2000 (Thermo Scientific, Waltham, MA, USA). RNA integrity was evaluated using an Agilent 2100 Bioanalyzer (Agilent, Santa Clara, CA, USA). After the samples were qualified, the library was purified and recovered, the sticky end was repaired, and the cDNA library was constructed by PCR amplification. The library construction and sequencing were performed by Personalbio Technology Co., Ltd. (Shanghai, China). Clean reads were generated through Cutadapt (v1.15) software, which eliminated data with adapters and low-quality reads (average quality below Q20). These high-quality clean reads were aligned with the rat genome reference genome using HISAT2 v2.0.5. DESeq2 (v1.4.5) was used to identify differentially expressed genes (DEGs) which were defined as genes meeting the conditions of false discovery rate (FDR) < 0.05 and |log2FoldChange (FC)| > 1. In addition, the annotated genes were further analyzed using Gene Ontology (GO) and Kyoto Encyclopedia of Genes and Genomes (KEGG) enrichment analyses. Functional enrichment analyses, including GO and KEGG Pathway analyses, were performed on the gene set using the Personalbio Cloud Platform (https://cloud.majorbio.com/). Statistical significance for pathway enrichment was defined as *p* < 0.05.

### 2.4. Metabolomics Analysis

#### 2.4.1. Sample Preparation

Cells were cultured in 10 cm^2^ dishes and harvested when reaching a density of ≥1 × 10^7^ cells. The culture medium was aspirated, followed by 2–3 washes with ice-cold PBS. Residual PBS was completely removed. Subsequently, 1 mL of pre-chilled LC-MS grade methanol/acetonitrile/water (2:2:1, *v*/*v*/*v*) was added for metabolite extraction. Cells were immediately scraped using a sterile cell scraper and transferred to pre-chilled polypropylene centrifuge tubes. Tubes were flash-frozen in liquid nitrogen and stored at −80 °C until LC-MS analysis.

#### 2.4.2. LC-MS/MS Conditions

The metabolomic profiles of the cells in the different treatment groups (*n* = 6) were characterized using LC-MS/MS with the assistance of Personalbio Technology Co., Ltd. (Shanghai, China). The LC analysis was performed on a Vanquish UHPLC system (Thermo Fisher Scientific, Waltham, MA, USA) using an ACQUITY UPLC^®^ HSS T3 column (2.1 × 100 mm, 1.8 µm) (Waters, Milford, MA, USA) that was maintained at 40 °C. The flow rate and injection volume were set at 0.3 mL/min and 2 μL, respectively. For LC-ESI (+)-MS, the mobile phases consisted of 0.1% (*v*/*v*) formic acid in acetonitrile (B2) and 0.1% (*v*/*v*) formic acid in water (A2). Separation was achieved with the following gradient: 0–1 min, 10% B2; 1–5 min, 10–98% B2; 5–6.5 min, 98% B2; 6.5–6.6 min, 98–10% B2; and 6.6–8 min, 10% B2. For LC-ESI (−)-MS, the analytes were dissolved in acetonitrile (B3) and 5 mM ammonium formate (A3), and separation was achieved with the following gradient: 0–1 min, 10% B3; 1–5 min, 10–98% B3; 5–6.5 min, 98% B3; 6.5–6.6 min, 98–10% B3; and 6.6–8 min, 10% B3 [19]. MS detection of the metabolites was performed on an Orbitrap Exploris 120 device (Thermo Fisher Scientific) with an ESI ion source. Simultaneous MS1 and MS/MS (full MS-ddMS2 mode, data-dependent MS/MS) acquisition was used. The parameters were as follows: sheath gas pressure, 40 arb; aux gas flow, 10 arb; spray voltage, 3.50 kV for ESI(+) and −2.50 kV for ESI (−); capillary temperature, 325 °C; MS1 range, *m*/*z* 100–1000; MS1 resolving power, 60,000 FWHM; number of data-dependent scans per cycle, 4; MS/MS resolving power, 15,000 FWHM; normalized collision energy, 30%; dynamic exclusion time, automatic [20].

#### 2.4.3. Metabolomics Data Processing and Analysis

The raw MS files were converted into mzXML format using the MSConvert tool within the ProteoWizard software package (v3.0.8789). The detection, filtering, and alignment of the peaks were performed using the XCMS package (v3.12.0) with the following parameter settings: bw = 2, ppm = 15, peakwidth = c (5, 30), mzwid = 0.015, mzdiff = 0.01, and method = centWave, yielding a quantitative list of metabolites. Subsequently, data correction was implemented via normalization of the total peak area to eliminate systematic errors. To obtain qualitative metabolite results, metabolite identification was carried out by searching against public spectral databases, including the Human Metabolome Database (HMDB) (http://www.hmdb.ca), MassBank (http://www.massbank.jp/), LipidMaps (http://www.lipidmaps.org), mzCloud (https://www.mzcloud.org), and KEGG (https://www.genome.jp/kegg/), as well as the company’s in-house database of standards.

Multivariate statistical analyses included principal component analysis (PCA) and orthogonal partial least squares discriminant analysis (OPLS-DA). Variable Importance for the Projection (VIP) values were obtained from the OPLS-DA model, and the contribution of each variable to classification was evaluated by VIP in prediction. Differentially expressed metabolites (DEMs) were considered statistically significant with *p* < 0.05, fold change > 1.5, and VIP > 1, and Student’s *t*-test was applied to measure the significance of each metabolite. Additionally, KEGG analysis was performed on the metabolites, and further analysis of metabolite-enriched pathways was conducted in the MetaboAnalyst 5.0 software.

### 2.5. Quantitative Real-Time PCR Analysis

To validate the reliability of RNA-Seq results, 12 genes related to the transcriptome results were selected for fluorescence quantitative PCR (qPCR) reactions. The real-time fluorescence quantitative analyzer (LightCycler480II, 384, Roche, Mannheim, Germany) was used, with gapdh as the internal reference gene. The primer information used in the experimental procedure is shown in Supplementary File S3: Table S2. All samples were set with three replicates, and the internal reference gene was used to normalize sample variations. After comparing samples with the CK group, the gene expression levels were represented by the 2^−∆∆Ct^ method.

### 2.6. Detection of Cell Cycle

After starvation, all cells were divided into four groups: CK group, LPS group, LPS + S50 low-dose group (1 mg/mL) (LPS + S50(L)), and LPS + S50 high-dose group (2 mg/mL) (LPS + S50(H)). Following the procedures described in Section 2.2, subsequent experimental assays were performed. The cell cycle was analyzed using a kit from Beyotime Biotech Inc. (Shanghai, China). Propidium iodide (PI) staining was employed to measure DNA content, using a flow cytometer (BD FACSAria III, DeLand, FL, USA).

### 2.7. Intercellular ATP, NAD^+^/NADH Ratio Measurements

The cellular ATP, NAD^+^, and NADH levels were measured using ultraviolet spectrophotometry at wavelengths of 340 nm, 570 nm, and 570 nm, respectively, following the protocol specified in the assay kit (Solarbio Science & Technology Co., Ltd., Beijing, China).

### 2.8. Measurement of Intracellular Ca^2+^ Level

The intracellular Ca^2+^ concentration was measured using Fluo-3 AM as the fluorescent probe, following the protocol provided in the Solarbio Science & Technology Co., Ltd. (Beijing, China) assay kit. Briefly, cells from each group were adjusted to a density of 1 × 10^6^ cells/mL, and a 5 μM Fluo-3 AM working solution was prepared using Fluo-3 AM/DMSO solution and Pluronic F127 solution. The working solution was added to the cells, followed by incubation at 37 °C for 20 min. Subsequently, a 5× volume of HBSS containing 1% FBS was added, and the cells were further incubated for 40 min. Finally, the cells were washed and resuspended in HEPES-buffered saline before analysis. The intracellular Ca^2+^ was measured with flow cytometry (BD FACSAria III, DeLand, FL, USA) at λex 506 nm and λem 525 nm. The mean values of fluorescence intensity were also calculated.

### 2.9. Statistical Analysis

All data were represented and calculated from at least three replicates and are expressed as the mean ± standard deviation (SD). One-way analysis of variance (ANOVA) using a general linear model followed by multiple comparisons using the Tukey method post hoc test was performed using GraphPad Prism9 (GraphPad Softward, Inc., San Diego, CA, USA). Significant differences were determined by a *p* value of less than 0.05 (two-tailed).

## 3. Results

### 3.1. Transcriptome Analysis

#### 3.1.1. The Results of RNA Sequencing Reads

**Figure 1:**
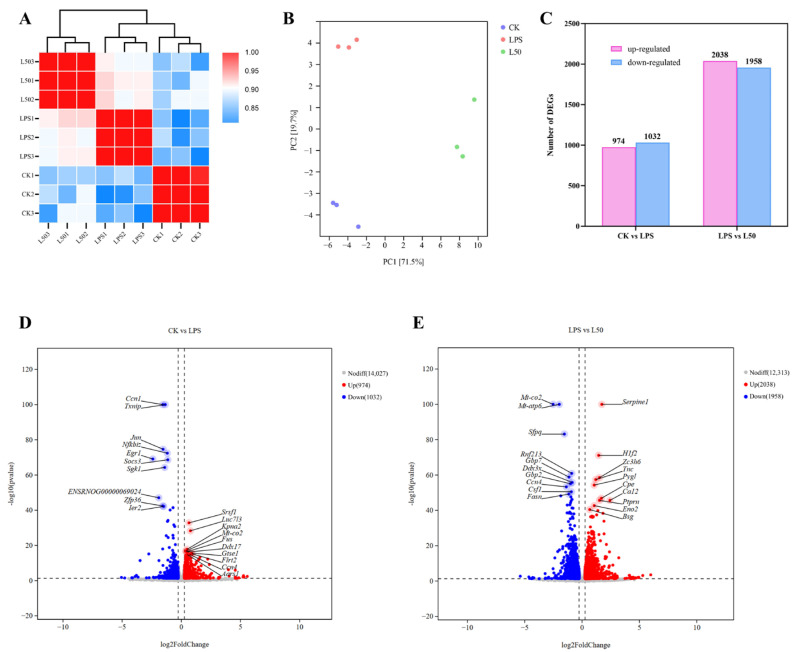
Correlation analysis of different group samples (**A**), principal component analysis of samples (**B**), bar graph showing the number of up-regulated and down-regulated DEGs (**C**), volcano plot of up-regulated and down-regulated DEGs in the CK vs. LPS (**D**) and LPS vs. L50 comparisons (**E**).

We sequenced and assembled the transcriptomic cells after group treatment and compared them with the cells without any treatment. A total of 388,425,496 raw reads and 383,612,536 high quality clean reads from each library were obtained. Furthermore, the percentage of Q30 bases in each sample is more than 95% and the GC content was at least 48.44%. The details are shown in Supplementary File S3: Table S3. Supplementary File S3: Table S4 showed that 96.59–96.88% of the sequences matched a unique position to the rat genome. All the statistics indicated that the quality of RNA sequencing reads met the requirements for the subsequent bioinformatics analysis. Further, Pearson’s correlation coefficient exhibited in Figure 1A suggested that there was a high correlation among the biological replicates of different groups and that different treatments affected the transcriptome of cells. The PCA plot (Figure 1B) indicates that there are significant overall differences among the three sample groups, with PCA1 and PCA2 accounting for 71.5% and 19.7% of the variance, respectively. Figure 1C shows the DEGs found in the CK vs. LPS and LPS vs. L50 groups. Under the filter criteria of a *p*-value < 0.05 and |log_2_FoldChange (FC)| > 1, a total of 2006 DEGs were identified in the CK vs. LPS group, with 974 upregulated and 1032 downregulated. In the LPS vs. L50 group, a total of 3996 DEGs were identified, with 2038 upregulated and 1958 downregulated. Two volcano plots (Figure 1D,E) show the overall differential expression status of genes in two comparative groups. Red indicates upregulated genes, blue indicates downregulated genes, and gray indicates non-significantly DEGs. The gene names marked with names are the top 10 up and downregulated genes.

#### 3.1.2. GO and KEGG Enrichment Analysis of DEGS

**Figure 2:**
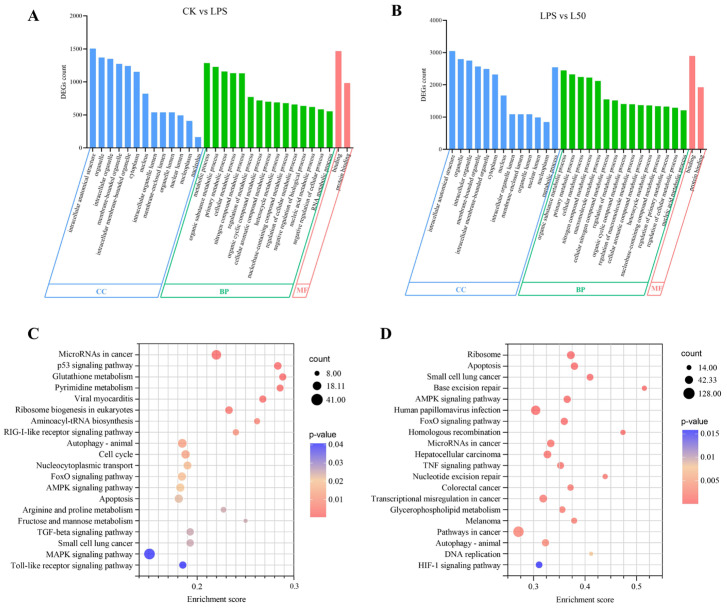
Enriched Go terms for DEGs of CK vs. LPS (**A**) and LPS vs. L50 (**B**). Results are summarized for the three main GO categories, including biological process (BP), cellular component (CC), and molecular function (MF). KEGG enrichment classification of DEGs of CK vs. LPS (**C**) and LPS vs. L50 (**D**).

The top 30 significant GO analysis results for the two comparative groups are shown in Figure 2A,B. In the CC categories, the DEGs in the CK vs. LPS and LPS vs. L50 comparative groups are mainly enriched in intracellular anatomical structure, organelle, and intracellular organelle. In the BP categories, besides the two comparative groups both significantly enriched in the organic substance metabolic process and primary metabolic process, the DEGs in CK vs. LPS are enriched in the metabolic process, while those in LPS vs. L50 are enriched in the cellular metabolic process. For the enrichment results of MF, the DEGs in both groups are mainly expressed in the function of binding and protein binding.

KEGG enrichment pathway analysis was applied to explore the functional implications of DEGs in the cells that have been treated differently. Using a *p* value threshold of <0.05 analyzed the results of the KEGG enrichment analysis, and the top 20 enrichment pathways were selected for each comparison group, as shown in Figure 2C,D. The results indicate that both the CK vs. LPS group and the LPS vs. L50 group exhibited significant enrichment in four pathways: AMPK signaling pathway, Apoptosis, FoxO signaling pathway, and Autophagy—animal. These findings suggest that SZS extract may mitigate LPS-induced cellular damage by regulating apoptosis, modulating the AMPK/FoxO signaling axis, and enhancing cell growth and survival.

As shown in Supplementary File S3: Table S5, after removing duplicate genes, a total of 32 DEGs were identified in both comparison groups. These DEGs were co-modulated by LPS and SZS extract in the AMPK signaling pathway, apoptosis, FoxO signaling pathway, and autophagy-animal, exhibiting opposite regulatory trends.

#### 3.1.3. Identification of Potential Target Genes

Due to the complex interactions among these overlapping genes, their interrelationships could not be intuitively discerned. Therefore, we constructed a protein–protein interaction (PPI) network comprising all 32 overlapping genes to further investigate their roles in biological signal transduction and gene expression regulation.

**Figure 3:**
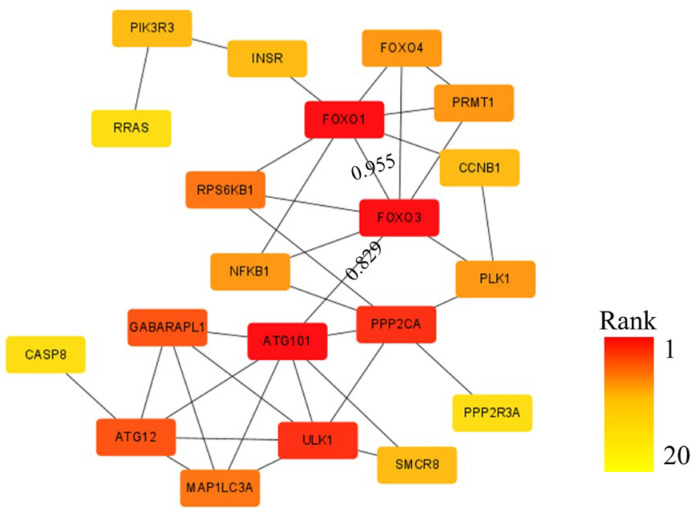
Protein–protein interaction (PPI) network constructed using DEGs in the overlapping pathways of two comparison groups in transcriptomics.

Using the STRING database and Cytoscape 3.10.2 software with a minimum required interaction score set at high confidence (0.700), we generated the PPI network which is shown in Figure 3. The network consists of 31 nodes and 39 connecting edges. In PPI networks, node degree (the number of edges connected to a node) reflects its functional importance, with higher-degree nodes playing more crucial roles. Notably, ATG101, FOXO1, and FOXO3 exhibited the highest node degree of 7, identifying them as hub nodes that participate in multiple biological processes.

Particularly, FOXO3 served as a central connector gene, demonstrating the highest binding scores with both ATG101 (0.829) and FOXO1 (0.995). Collectively, our findings identify FOXO3 as a master regulator that orchestrates cross-pathway communication, with its resident AMPK pathway forming the backbone of the entire regulatory network.

#### 3.1.4. Regulation the Pathway of AMPK Signaling Pathway

**Figure 4:**
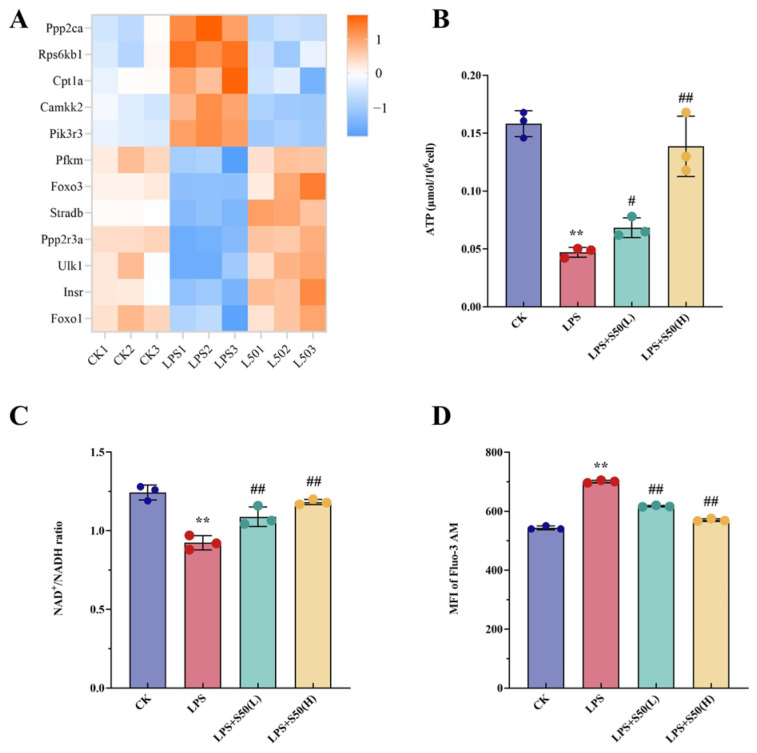
Heat maps of the expression profiles of 12 DEGs involved in the AMPK signaling pathway (**A**), different treatments affect the endogenous ATP content (**B**), NAD^+^/NADH content (**C**) and intracellular Ca^2+^ levels detected by flow cytometry (**D**). The vertical bars indicate standard deviation (*n* = 3). Data are presented as the mean ± SD. Asterisks indicate statistically significant differences between CK and LPS (**, *p* < 0.01). Pound signs indicate statistically significant differences between LPS and L50 (^#^, *p* < 0.05; ^##^, *p* < 0.01).

As illustrated in Figure 4A, the AMPK signaling pathway involved alterations in 12 genes, including 5 significantly upregulated genes and 7 significantly downregulated genes after LPS treatment. Notably, SZS extract co-treatment effectively reversed all these LPS-induced alterations. To further validate the effects of exogenous treatments on key products in the AMPK signaling pathway, we measured intracellular ATP levels, NAD^+^/NADH ratio, and calcium ion concentration. Figure 4B demonstrated that LPS treatment significantly decreased ATP levels compared to the CK group (*p* < 0.01), while both low and high doses of SZR extract effectively restored ATP content, with the high-dose group showing higher ATP levels (*p* < 0.01). As shown in Figure 4C, LPS treatment significantly reduced the NAD^+^/NADH ratio, which was markedly increased by SZS extract pretreatment. Figure 4D revealed that LPS exposure for 24 h elevated intracellular Fluo-3/AM fluorescence intensity to 1.3 times the CK value, whereas pretreatment with two concentrations of SZS extract for 1 h prior to LPS administration significantly reduced the fluorescence intensity by 14% and 19.6%, respectively.

#### 3.1.5. Effect of Different Treatment on the Cell Cycle

**Figure 5:**
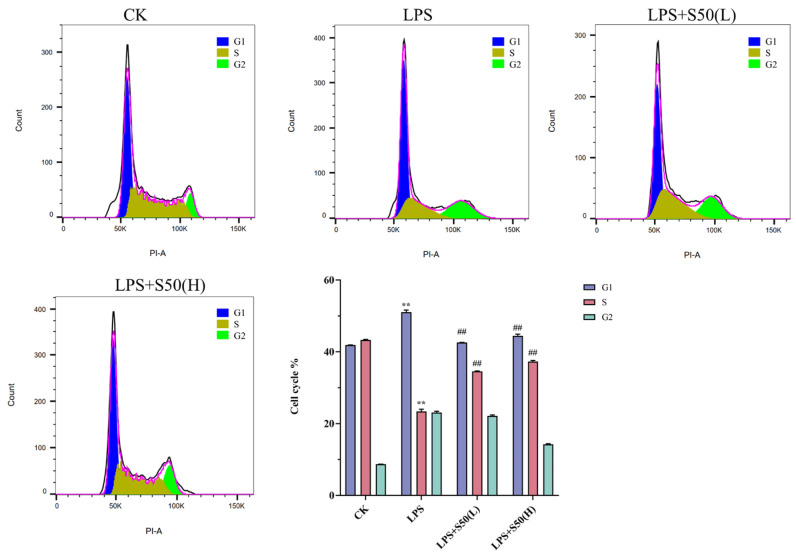
Different treatments affect cell cycle monitoring by flow cytometry with PI-staining. The vertical bars indicate standard deviation (*n* = 3). Data are presented as the mean ± SD. Asterisks indicate statistically significant differences between CK and LPS (**, *p* < 0.01). Pound signs indicate statistically significant differences between LPS and L50 (^##^, *p* < 0.01).

Cell cycle analysis via PI staining revealed distinct DNA content variations across different phases (Figure 5). Compared to the CK group, LPS treatment significantly altered cell cycle distribution with G1 and S phase proportions measuring 51.03 ± 0.63% (*p* < 0.05) and 23.04 ± 0.60% respectively, indicating LPS-induced G1/S phase arrest that impeded normal DNA replication. Dose-dependent administration of SZS extract progressively increased S phase cell populations, with S phase proportions reaching 34.60 ± 0.10% and 37.30 ± 0.30% (*p* < 0.05) at concentrations of 1 mg/L and 2 mg/L respectively. These findings demonstrate that SZR extract effectively alleviates LPS-mediated G1/S phase blockade and restores normal cell cycle progression.

### 3.2. qRT-PCR Verification

**Figure 6:**
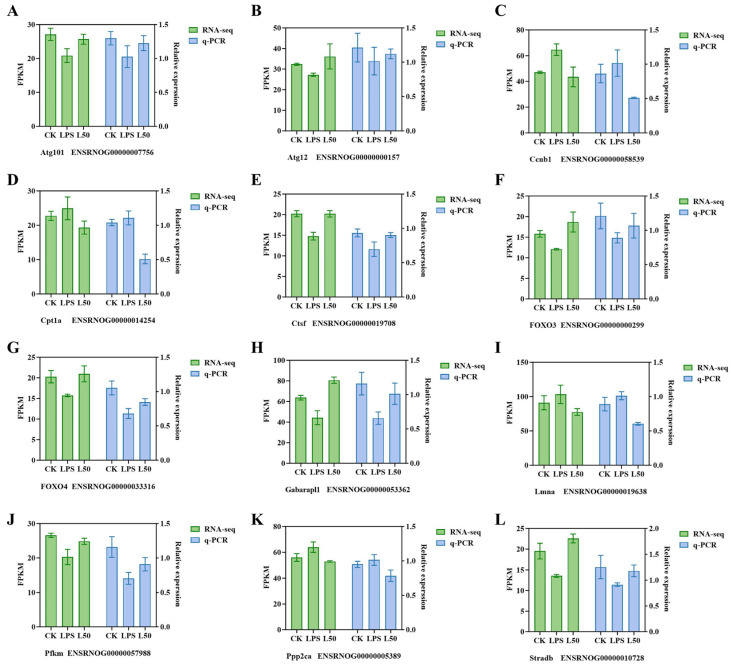
The changes in expression levels of 12 DEGs as determined by RNA-seq and qRT-PCR (**A**–**L**). Error bars represent the standard deviation of the means.

12 candidate genes (Figure 6) were selected from DEGs involved in the 4 related pathways, which were verified by qRT-PCR to establish the authenticity of the RNA-seq data. The relative expression of candidate DEGs was expressed as FPKM. The overall expression trend of candidate genes indicated that it was in accordance with the expression in the sequencing data, suggesting that the transcriptome sequencing data were sufficiently persuasive.

### 3.3. Metabolomic Analysis

#### 3.3.1. Multivariate Statistical Analysis

**Figure 7:**
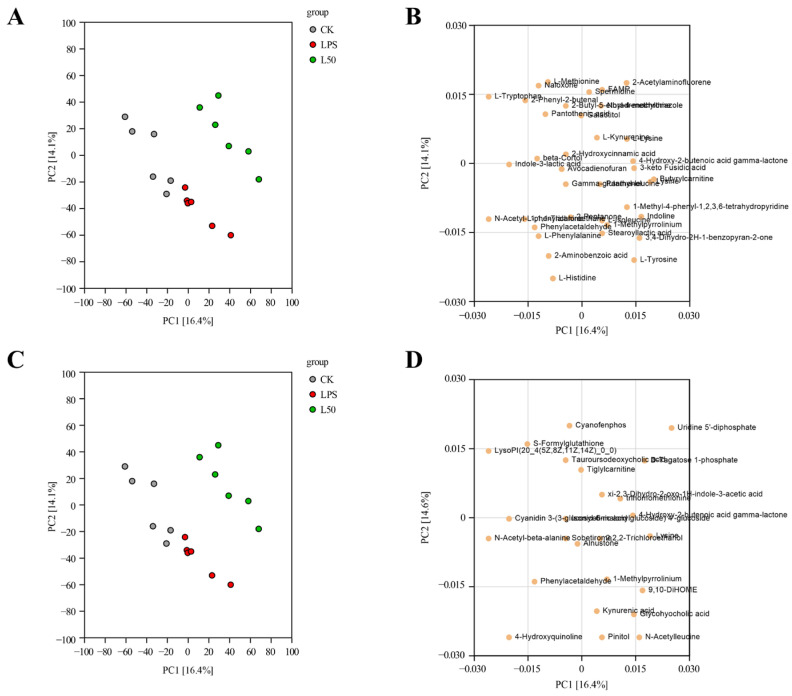
PCA scores and loading plots of the three different treatment groups: positive-ion mode (**A**,**B**); negative-ion mode (**C**,**D**). CK represents the control group, LPS represents the group treated with LPS, and L50 represents the group treated with LPS and 50% ethanol extract.

The graphs in Figure 7 display the overall PCA scores and loading plots of samples from the CK, LPS, and L50 groups in both positive- and negative-ion modes. The analysis revealed significant separation among the treatment groups, indicating distinct metabolic differences among them. As evident from the loading plot, multiple metabolites such as L-tryptophan, L-histidine, uridine 5′-diphosphate, and 4-hydroxyquinoline contributed to the inter-sample differences.

To further evaluate the discriminatory power between groups, OPLS-DA was performed. The results obtained under the positive- and negative-ion scanning modes (Supplementary File S2: Figure S1 and Supplementary File S3: Table S6) showed that the R^2^Y value was close to 1, indicating that the groups can be well distinguished and their metabolic profiles differ significantly. The Q^2^ values were all higher than 0.5, proving that the model is effective and can be used to screen potential biomarkers with statistical significance. Furthermore, after 200 permutation tests, no fit occurred in the model, indicating that the data are reliable.

#### 3.3.2. Determination of DEMs and Pathway Enrichment Analysis

**Figure 8:**
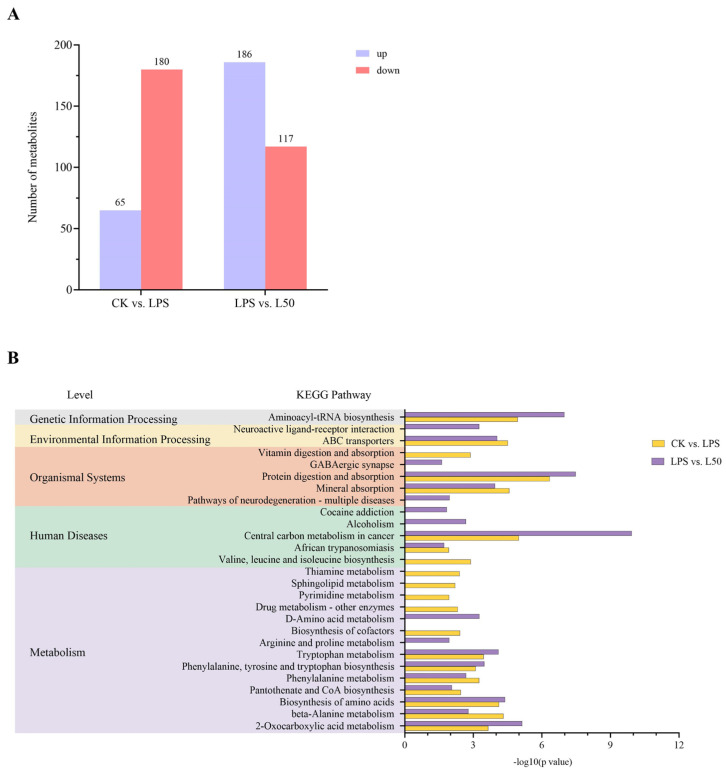
Bar graph showing the number of up-regulated and down-regulated DEMs (**A**), KEGG pathway enrichment of DEGs in different levels (**B**).

The VIP value is commonly employed to identify biologically significant DEMs, with VIP > 1 indicating variables of substantial importance. Accordingly, we established VIP > 1 combined with *p* value < 0.05 as the screening criteria for significant DEMs, conducting comprehensive screening in both positive and negative ion modes. As illustrated in the bar chart of Figure 8A compared with the CK group, the LPS group exhibited 245 DEMs (65 upregulated and 180 downregulated). In contrast, the L50 group displayed 303 DEMs relative to the LPS group (186 upregulated and 117 downregulated). Metabolites meeting the *p* < 0.05 threshold were visually represented through volcano plots (Supplementary File S2: Figure S2).

We performed KEGG enrichment analysis to further investigate the functional implications of DEMs, categorizing the enriched pathways into five major classes according to level 1 classification. Our results (Figure 8B) revealed that DEMs from both comparison groups were simultaneously enriched in the ABC transporters pathway under “Environmental Information Processing” and multiple metabolic pathways including 2-oxocarboxylic acid metabolism, beta-Alanine metabolism, and phenylalanine metabolism. Notably, DEMs from the SZS extract group showed additional enrichment in neuroactive ligand–receptor interaction and GABAergic synapse pathways, which are closely associated with neural signaling molecules. Table 1 presents key DEMs involved in these nervous system-related pathways from both comparison groups.

## 4. Discussion

**Figure 9:**
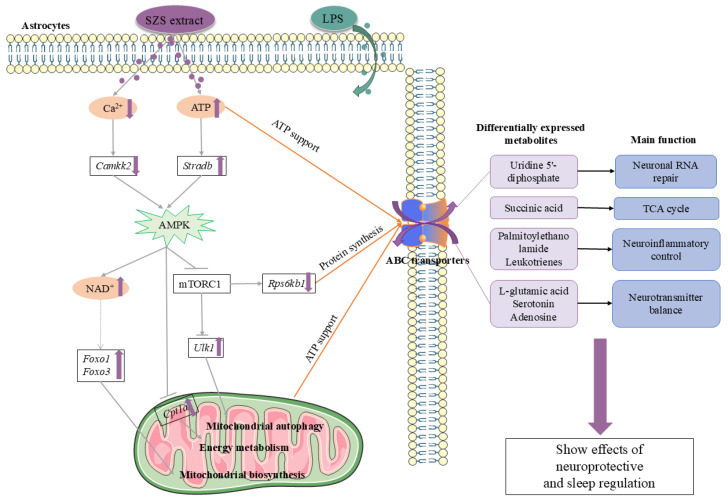
Hypothetical model of the molecular mechanism by which *Semen Ziziphi Spinosae* (SZS) extract activates the AMPK signaling pathway in LPS-treated astrocytes, thereby inducing energy metabolism, maintaining normal function of ABC transporters, and facilitating transmembrane transport of metabolites. “→” in grey indicated activation effect, while “⊥” in grey indicated inhibition effect. (Copyright Citation: Image provided by Servier Medical Art (https://smart.servier.com/), licensed under CC BY 4.0 (https://creativecommons.org/licenses/by/4.0/).

As a commonly used medicinal-food homologous plant for treating neurological disorders, SZS exhibits multiple neuroprotective properties, including sleep improvement, antidepressant effects, anxiolytic activity, and memory enhancement [21]. Astrocytes, constituting the largest non-neuronal cell population in the central nervous system, play pivotal roles in maintaining neuronal ion and energy metabolic homeostasis, regulating neurotransmitter levels, and modulating synaptic plasticity [22]. In the present study, we employed SZS extract as a therapeutic intervention against LPS-induced astrocyte injury, combining transcriptomics and metabolomics approaches to systematically analyze drug-treated astrocytes at both genetic and metabolic levels. Our findings demonstrate that SZS extract exerts a damage repair effect on LPS-injured astrocytes by restoring cellular energy balance, maintaining ABC transporter function, and regulating synthesis and release of neurofunction-related metabolites (Figure 9).

LPS not only triggers cellular damage through its classical inflammatory pathways but also induces apoptosis and cell cycle arrest while disrupting autophagy homeostasis [23]. Our transcriptomics results revealed that the differentially expressed genes (DEGs) in both the CK vs. LPS and LPS vs. L50 comparison groups showed significant enrichment in autophagy and apoptosis pathways, indicating that LPS threatens cell survival through these dual mechanisms. In the SZS extract-treated group, the upregulation of core autophagy genes ATG12 and ATG101 suggests that SZS extract may enhance protective autophagy to clear damaged organelles. At the same time, the significant downregulation of the casp8 gene expression blocks death receptor-mediated apoptosis initiation, thereby counteracting the pro-apoptotic effect of LPS [24]. Furthermore, using PI staining, we found that LPS induction significantly increased the proportion of G1 phase cells while markedly reducing S phase cells, indicating cell cycle arrest at the G1/S phase that impedes DNA synthesis and inhibits cell proliferation. However, pretreatment with SZS extract alleviated this G1/S phase blockade, allowing normal cell cycle progression and reducing astrocyte injury. These results suggest that SZS extract exerts protective effects against cellular damage and promotes survival.

In the transcriptomic analysis of this study, PPI network construction of DEGs from four enriched pathways revealed FOXO3 as the hub gene. As a member of the Forkhead transcription factor family, FOXO3 is a pivotal regulator of antioxidant defense, anti-inflammatory responses, and autophagy [25]. Our data demonstrated that LPS-induced downregulation of FOXO3 likely inhibited its phosphorylation and cytoplasmic translocation, thereby triggering an apoptosis proliferation imbalance and disrupting glucose/lipid metabolism. Notably, SZS extract treatment restored FOXO3 expression, potentially attributable to its active components (e.g., flavonoids) directly stimulating FOXO3 transcription. These findings identify FOXO3 as the key mediator of SZS’s protective effects against LPS-induced astrocyte injury, suggesting that the AMPK signaling pathway—where FOXO3 operates—may plays a central role in the therapeutic mechanism of SZS extract.

AMPK, a central regulator of cellular energy homeostasis, orchestrates downstream pathways by sensing AMP/ATP ratios and Ca^2+^ fluctuations to modulate glucose uptake, fatty acid oxidation, and mitochondrial function [26,27,28]. However, under pathological conditions (e.g., inflammation, diabetes, aging, and cancer), this regulatory network becomes compromised [29]. A previous findings demonstrating that LPS exposure reduces ATP levels [30], and elevates basal Ca^2+^ levels in rat primary astrocytes [31], thereby disrupting AMPK activation thresholds. Our study revealed analogous perturbations. Notably, SZS extract effectively counteracted LPS-induced ATP depletion and Ca^2+^ overload in astrocytes, suggesting its capacity to restore energy metabolism by recalibrating AMPK’s sensory function. Transcriptomic profiling identified 12 genes coordinately modulated by both LPS and SZS extract. Intriguingly, compared to LPS treatment alone, SZS supplementation downregulated the AMPK upstream kinase Camkk2 while upregulating Stradb, indicating its ability to reestablish AMPK-mediated gene expression and metabolic homeostasis. Within AMPK downstream pathways, SZS extract significantly rescued the LPS-induced decline in NAD^+^/NADH ratios. As an essential cofactor for sirtuins (e.g., SIRT1), NAD^+^ activates FOXO transcription factors to promote mitochondrial biogenesis (e.g., via PGC-1α upregulation) and antioxidant defenses [32,33]. Moreover, NAD^+^ restoration directly correlated with enhanced electron transport chain activity, thereby supporting ATP formatted by ATP synthase complex [34,35]. These findings align with recent research demonstrating that jujuboside A, a major active constituent of SZS, enhances ATPase activity, ATP production, and mitochondrial function in prefrontal neurons of insomnia model mice [36]. Based on these findings, we propose that SZS extract may function as an AMPK activator that either directly stimulates AMPK or amplifies its upstream signaling to ameliorate LPS-induced disturbances in astrocytic energy metabolism and restore mitochondrial function.

ABC transporters constitute a ubiquitous superfamily of membrane proteins that facilitate the transmembrane translocation of diverse substrates by utilizing the chemical energy derived from ATP hydrolysis. These critical transporters participate in numerous physiological processes, yet their expression and function in brain microglia are significantly compromised during LPS-induced neuroinflammation [37]. Our metabolomic analysis revealed the abnormal distribution of ABC transporter-related metabolites (including L-Serine and Sucrose) in LPS-treated astrocytes, indicating functional impairment of these transport systems. The operational efficacy of ABC transporters depends not only on ATP hydrolysis but also on dynamic protein expression regulation. As previously demonstrated, SZS extract exhibits a remarkable capacity to activate AMPK signaling, restore LPS-induced ATP depletion, and promote mitochondrial biogenesis. Furthermore, as a central energy sensor, AMPK can directly modulate ABC transporter activity through phosphorylation modifications. Notably, studies have demonstrated that curcumin upregulates ABCA1 and ABCG1 mRNA expression in THP-1 human macrophages by activating the AMPK-liver X receptor α (LXRα) signaling pathway, thereby exerting its cholesterol-lowering effects [38]. Additionally, AMPK can indirectly regulate ABC transporter gene expression by either activating transcription factors (including SREBP1c, FOXO, and PGC-1α) or suppressing mTORC1 activity [39]. Our study identified that SZS extract normalizes LPS-altered INSR and PIK3R3 gene expression, subsequently modulating mTORC1 activity and enhancing Rps6kb1 (S6K-encoding gene) phosphorylation. This cascade potentially influences ABC transporter biosynthesis, given S6K’s established role in promoting protein synthesis and cellular growth. These findings illuminate a dual regulatory mechanism whereby AMPK maintains ABC transporter function through energy homeostasis and transcriptional control. The therapeutic potential of SZS extract appears to stem from its ability to leverage this AMPK-ABC transporter axis, thereby preserving metabolic homeostasis in LPS-challenged astrocytes.

Our metabolomic analysis uncovered that SZS extract not only modulates ABC transporter activity but also exerts broad regulatory effects on neural metabolic networks under the LPS challenge. Notably, these effects were particularly evident in the neuroactive ligand receptor interaction and GABAergic synapse pathways—both critically involved in neurotransmitter homeostasis. As key regulators of central nervous system homeostasis, astrocytes play pivotal roles in maintaining neurotransmitter balance, energy metabolism, and neuroinflammatory control [40,41], all of which are closely associated with insomnia pathophysiology. In this study, SZS treatment significantly increased uridine 5′-diphosphate levels, a key precursor for pyrimidine nucleotide synthesis that facilitates neuronal RNA repair, while modulating neurotransmitter profiles by elevating serotonin and reducing L-glutamate and adenosine levels, suggesting enhanced serotonergic signaling and attenuated excitotoxicity. The extract also suppressed proinflammatory mediators including leukotrienes and palmitoylethanolamide, while reducing succinic acid levels. As a key intermediate in the TCA cycle, the decrease in succinate is likely associated with its involvement in this metabolic pathway [42]. These findings are consistent with our previous demonstration of SZS’s ability to maintain energy homeostasis by preserving mitochondrial function. ABC transporters mediate diverse transmembrane transport processes, with inward transporters facilitating cellular uptake of nutrients like amino acids and sugars to support cellular growth, while outward transporters eliminate xenobiotics and metabolic byproducts to maintain low intracellular concentrations of potentially harmful substances [43]. These collective findings demonstrate that SZS extract coordinately regulates metabolite transport, neurotransmitter balance, and lipid-mediated inflammatory responses, with its beneficial effects on astrocyte-mediated sleep regulation likely being closely associated with its modulation of ATP-binding cassette transporters.

However, this study lacks direct experimental confirmation that the gene-level changes (e.g., in FOXO3/AMPK) caused the observed changes in metabolite levels (e.g., ABC transporter, neurotransmitter). Although our discussion outlines plausible associations within the proposed FOXO3-energy metabolism-ABC transporter axis, this hypothesis requires further validation. For instance, using AMPK activators or inhibitors in combination with SZS would clarify the precise role of AMPK signaling in this cascade. Similarly, FOXO3-specific siRNA or inhibitors can be used to determine whether the beneficial effects of SZS on energy metabolites (e.g., ATP, NAD^+^) and ABC transporter function are FOXO3 dependent. Furthermore, the use of conditional FOXO3-knockout mice would provide a more direct means to validate the role of the axis in insomnia-related phenotypes, neurotransmitter levels, and cellular gene expression.

## 5. Conclusions

Our integrated transcriptomic and metabolomic analyses demonstrate that SZS extract ameliorates LPS-induced astrocyte injury primarily through a regulatory network with FOXO3 as center, while concomitantly restoring intracellular ATP and Ca^2+^ homeostasis to modulate upstream AMPK signaling. Furthermore, SZS treatment enhances cellular energy provision for ABC transporters, thereby influencing the secretion of neurofunction-related metabolites. These findings collectively validate the therapeutic efficacy of SZS extract in restoring dysfunctional astrocytes. The observed multi-target and multi-pathway regulatory mechanism exemplifies the characteristic holistic therapeutic approach of traditional Chinese medicine, providing novel mechanistic insights into the potential application of SZS for insomnia management.

## Figures and Tables

**Table 1:** Differential metabolites related to nervous system pathways.

Pathway Name	CK vs. LPS			LPS vs. L50		
	*p* Value	Metabolites		*p* Value	Metabolites	
		Up-Regulated	Down-Regulated		Up-Regulated	Down-Regulated
ABC transporters	0.0000308	L-Serine; Sucrose; Biotin; Uridine; Cytidine; Iron	L-Lysine; L-Phenylalanine; L-Histidine; Spermidine; L-Isoleucine;	0.0000905	L-Lysine; L-Arginine; L-Phenylalanine; L-Histidine; L-Proline; L-Valine; Spermidine; L-Isoleucine; Xanthosine; Deoxyinosine;	L-Glutamic acid; Adenosine
Neuroactive ligand receptor interaction	-	-		0.000552	Uridine 5′-diphosphate; Serotonin	L-Glutamic acid; Adenosine; Leukotriene; Palmitoylethanolamide
GABAergic synapse	-	-		0.023978	-	L-Glutamic acid; Succinic acid

## Data Availability

The data presented in this study are available on request from the corresponding author due to the data that has been used is confidential.

## Supplementary Materials

The following supporting information can be downloaded at: https://www.mdpi.com/article/10.3390/nu17223498/s1, Supplementary File S1: Supplementary Method and Table S1: Effect of S50 on CTX-TNA2 cells viability; Supplementary File S2: Figure S1: OPLS-DA score plots and permutation test results of different groups. Under positive ion mode (A,C); under negative ion mode (B,D); Figure S2: Volcano maps display the differential metabolites in LPS treatment group (LPS) compared with the control group under positive ion mode (A); the LPS group compared with the control group under negative ion mode (B); the LPS group compared with the LPS + 50% ethanol extract group (L50) under positive ion mode (C); the LPS group compared with L50 group under negative ion mode (D); Supplementary File S3: Table S2: Primer information for the experimental process; Table S3: RNA sequencing statistics of clean sequencing data; Table S4: Statistics on the alignment of clean reads and reference genomes; Table S5: Overlapping Genes in Four Pathways Between Two Comparative Groups; Table S6. The parameters of OPLS-DA analysis. Ref. [44] is cited in the supplementary materials.

## References

[1] Dressle R.J., Spiegelhalder K., Schiel J.E., Benz F., Johann A., Feige B., Jernelöv S., Perlis M., Riemann D. (2025). The Future of Insomnia Research-There’s Still Work to Be Done. J. Sleep Res..

[2] Lee Y., Lee S., Chang S.C., Lee J. (2019). Significant roles of neuroinflammation in Parkinson’s disease: Therapeutic targets for PD prevention. Arch. Pharmacal Res..

[3] Sheng F.Y., Zhang L.L., Wang S.S., Yang L.L., Li P. (2020). Deacetyl Ganoderic Acid F Inhibits LPS-Induced Neural Inflammation via NF-κB Pathway Both In Vitro and In Vivo. Nutrients.

[4] Xi X., Toth L.A. (2000). Lipopolysaccharide effects on neuronal activity in rat basal forebrain and hypothalamus during sleep and waking. Am. J. Physiol. Regul. Integr. Comp. Physiol..

[5] Aghelan Z., Pashaee S., Abtahi S.H., Karima S., Khazaie H., Ezati M., Khodarahmi R. (2023). Natural Immunosuppressants as a Treatment for Chronic Insomnia Targeting the Inflammatory Response Induced by NLRP3/caspase-1/IL1ß Axis Activation: A Scooping Review. J. Neuroimmune Pharmacol..

[6] Carvalhas-Almeida C., Serra J., Moita J., Cavadas C., Alvaro A.R. (2023). Understanding neuron-glia crosstalk and biological clocks in insomnia. Neurosci. Biobehav. Rev..

[7] Palagini L., Geoffroy P.A., Miniati M., Perugi G., Biggio G., Marazziti D., Riemann D. (2022). Insomnia, sleep loss, and circadian sleep disturbances in mood disorders: A pathway toward neurodegeneration and neuroprogression? A theoretical review. CNS Spectr..

[8] Wu R., Tripathy S., Menon V., Yu L., Buchman A.S., Bennett D.A., De Jager P.L., Lim A.S.P. (2023). Fragmentation of rest periods, astrocyte activation, and cognitive decline in older adults with and without Alzheimer’s disease. Alzheimers Dement..

[9] Eldridge S.L.S., Teetsel J.F.K., Torres R.A., Ulrich C.H., Shah V.V., Singh D., Zamora M.J., Zamora S., Sater A.K. (2022). A Focal Impact Model of Traumatic Brain Injury in *Xenopus* Tadpoles Reveals Behavioral Alterations, Neuroinflammation, and an Astroglial Response. Int. J. Mol. Sci..

[10] Wei Y.H., Zhang Y.Q., Sun J., Li W., Zhao X.T., Tian N., Cao Y.X., Xie J.B. (2024). Modulation of the receptor for advanced glycation end products pathway by natural polyphenols: A therapeutic approach to neurodegenerative diseases. Food Biosci..

[11] Hu N., Zeng C., Cao Y., Li X.H., Bai F., Wang J.H., Yang B.F., Li C.L. (2025). Therapeutic potential of Shilong Qingxue Granule and its extract against glutamate induced neural injury: Insights from in vivo and in vitro models. J. Ethnopharmacol..

[12] Janda K., Wojtkowska K., Jakubczyk K., Antoniewicz J., Skonieczna-Zydecka K. (2020). *Passiflora incarnata* in Neuropsychiatric Disorders—A Systematic Review. Nutrients.

[13] Ma J., Huang S., Shi L., Shen Y., Gao S., Wu Z. (2024). Research progress on the effect of medicine and food homology resources for sleep improvement. Heliyon.

[14] Fan W.X., Lu X.F., Meng X.L., Lan Y.R., Song B.Z., Ma Q.L., Chang L.P. (2014). Thermal Decomposition Mechanism and Kinetic Parameters of Semen Ziziphi Spinosae Based on Thermogravimetric Analysis. Asian J. Chem..

[15] Wang D.D., Ho C.T., Bai N.S. (2022). Ziziphi Spinosae Semen: An updated review on pharmacological activity, quality control, and application. J. Food Biochem..

[16] Bi A.Q., Liu R.H., Xie M., He B.S., Yan T.X., Du Y.Y., Jia Y. (2025). Semen Ziziphi Spinosae alleviates cardiomyocyte apoptosis in rats with coronary heart disease via the AMPK/SIRT1/PGC-1α signaling pathway activation. Phytomedicine.

[17] Yang T.T., Peng Y.T., Shao Y.T., Pan D.D., Cheng Q., Jiang Z.Z., Qian S.T., Li B.J., Yan M., Zhu X. (2025). Mitochondria-dependent apoptosis was involved in the alleviation of Jujuboside A on diabetic kidney disease-associated renal tubular injury via YY1/PGC-1α signaling. Phytomedicine.

[18] Park S.M., Lee T.H., Zhao R.J., Kim Y.S., Jung J.Y., Park C.A., Jegal K.H., Ku S.K., Kim J.K., Lee C.W. (2018). Amelioration of inflammatory responses by Socheongryong-Tang, a traditional herbal medicine, in RAW 264.7 cells and rats. Int. J. Mol. Med..

[19] Zelena E., Dunn W.B., Broadhurst D., Francis-McIntyre S., Carroll K.M., Begley P., O’Hagan S., Knowles J.D., Halsall A., Wilson I.D. (2009). Development of a Robust and Repeatable UPLC-MS Method for the Long-Term Metabolomic Study of Human Serum. Anal. Chem..

[20] Want E.J., Masson P., Michopoulos F., Wilson I.D., Theodoridis G., Plumb R.S., Shockcor J., Loftus N., Holmes E., Nicholson J.K. (2013). Global metabolic profiling of animal and human tissues via UPLC-MS. Nat. Protoc..

[21] Zhang M., Liu J.R., Zhang Y.Q., Xie J.B. (2022). Ziziphi Spinosae Semen: A Natural Herb Resource for Treating Neurological Disorders. Curr. Top. Med. Chem..

[22] Chen Y.C., Hu J.Y., Zhang Y., Peng L.L., Li X.Y., Li C., Wu X.Y., Wang C. (2026). Epilepsy therapy beyond neurons: Unveiling astrocytes as cellular targets. Neural Regen. Res..

[23] John A., Raza H. (2021). Azadirachtin Attenuates Lipopolysaccharide-Induced ROS Production, DNA Damage, and Apoptosis by Regulating JNK/Akt and AMPK/mTOR-Dependent Pathways in Rin-5F Pancreatic Beta Cells. Biomedicines.

[24] Leverrier S., Salvesen G.S., Walsh C.M. (2011). Enzymatically active single chain caspase-8 maintains T-cell survival during clonal expansion. Cell Death Differ..

[25] Rong X., Xu J., Jiang Y., Li F., Chen Y.L., Dou Q.P., Li D.P. (2021). Citrus peel flavonoid nobiletin alleviates lipopolysaccharide-induced inflammation by activating IL-6/STAT3/FOXO3a-mediated autophagy. Food Funct..

[26] Park J.M., Lee D.H., Kim D. (2023). Redefining the role of AMPK in autophagy and the energy stress response. Nat. Commun..

[27] Rabinovitch R.C., Samborska B., Faubert B., Ma E.H., Gravel S.P., Andrzejewski S., Raissi T.C., Pause A., St-Pierre J., Jones R.G. (2017). AMPK Maintains Cellular Metabolic Homeostasis through Regulation of Mitochondrial Reactive Oxygen Species. Cell Rep..

[28] Steinberg G.R., Hardie D.G. (2023). New insights into activation and function of the AMPK. Nat. Rev. Mol. Cell Biol..

[29] Canbolat E., Cakiroglu F.P. (2023). The importance of AMPK in obesity and chronic diseases and the relationship of AMPK with nutrition: A literature review. Crit. Rev. Food Sci. Nutr..

[30] Shin C.Y., Choi J.W., Ryu J.R., Ryu J.H., Kim W., Kim H., Ko K.H. (2001). Immunostimulation of rat primary astrocytes decreases intracellular ATP level. Brain Res..

[31] Birla H., Xia J.S., Gao X.H., Zhao H., Wang F.Y., Patel S., Amponsah A., Bekker A., Tao Y.X., Hu H.J. (2022). Toll-like receptor 4 activation enhances Orai1-mediated calcium signal promoting cytokine production in spinal astrocytes. Cell Calcium.

[32] Gupta S., Afzal M., Agrawal N., Almalki W.H., Rana M., Gangola S., Chinni S.V., KumarK B., Ali H., Singh S.K. (2025). Harnessing the FOXO-SIRT1 axis: Insights into cellular stress, metabolism, and aging. Biogerontology.

[33] Karagöz M.F., Celep A.G.S. (2023). The effect of caloric restriction on genetical pathways. Food Sci. Hum. Wellness.

[34] Luengo A., Li Z.Q., Gui D.Y., Sullivan L.B., Zagorulya M., Do B.T., Ferreira R., Naamati A., Ali A., Lewis C.A. (2021). Increased demand for NAD^+^ relative to ATP drives aerobic glycolysis. Mol. Cell.

[35] Schertl P., Braun H.P. (2014). Respiratory electron transfer pathways in plant mitochondria. Front. Plant Sci..

[36] Zhang Z., Che X., Feng T., Zou J., Chen G., Guo W., Ma C., Yuan H., Chen J., Xu X. (2025). Jujuboside A improves insomnia by maintaining mitochondrial homeostasis in prefrontal neurons. Brain Res. Bull..

[37] Gibson C.J., Hossain M.M., Richardson J.R., Aleksunes L.M. (2012). Inflammatory Regulation of ATP Binding Cassette Efflux Transporter Expression and Function in Microglia. J. Pharmacol. Exp. Ther..

[38] Sáenz J., Alba G., Reyes-Quiroz M.E., Geniz I., Jiménez J., Sobrino F., Santa-María C. (2018). Curcumin enhances LXRα in an AMP-activated protein kinase-dependent manner in human macrophages. J. Nutr. Biochem..

[39] Sahoo S., Kumari S., Neeli P.K., Pulipaka S., Kuncha M., Chandra Y., Annamaneni S., Kotamraju S. (2025). MTDHz7-mediated mTOR activation drives doxorubicin resistance in triple-negative breast cancer: Relevance of mTORC1 inhibition on chemosensitization. Cell. Signal..

[40] Andersen J.V., Schousboe A., Verkhratsky A. (2022). Astrocyte energy and neurotransmitter metabolism in Alzheimer’s disease: Integration of the glutamate/GABA-glutamine cycle. Prog. Neurobiol..

[41] Jing D.Q., Hou X.L., Guo X., Zhao X., Zhang K.X., Zhang J.W., Kan C.X., Han F., Liu J.L., Sun X.D. (2023). Astrocytes in Post-Stroke Depression: Roles in Inflammation, Neurotransmission, and Neurotrophin Signaling. Cell. Mol. Neurobiol..

[42] Raimundo N., Baysal B.E., Shadel G.S. (2011). Revisiting the TCA cycle: Signaling to tumor formation. Trends Mol. Med..

[43] Qu J., Chen T., Yao M., Wang Y., Xiao W., Li B. (2020). ABC transporter and its application in synthetic biology. Sheng Wu Gong Cheng Xue Bao Chin. J. Biotechnol..

[44] Meng L.S., Xing G., Li B., Li D.N., Sung X.Y., Yan T.C., Li L., Cao S., Meng X.J. (2018). Anthocyanins Extracted from *Aronia melanocarpa* Protect SH-SY5Y Cells against Amyloid-beta (1-42)-Induced Apoptosis by Regulating Ca^2+^ Homeostasis and Inhibiting Mitochondrial Dysfunction. J. Agric. Food Chem..

