# Supplementary material for: The Effects of Semen Ziziphi Spinosae Extract on LPS-Induced Astrocyte Gene Expression and Metabolites
Source: Nutrients. 2025 Nov 7;17(22):3498. doi: 10.3390/nu17223498 (PMC12655257; doi:10.3390/nu17223498)
Supplement: Supplementary file 1 [file nutrients-17-03498-s001.zip › Supplementary File S1.pdf]

Supplementary Method

Cell viability assay

Cells were seeded into 96-well plates at a density of 1×10^4 cells/well. achieving a seeding density of 1×10^4 cells/well. Following cell attachment, the culture medium was replaced with fresh medium containing SZS 50% ethanol extract (S50) at concentrations of 0, 0.01, 0.1, and 1 mg/mL, which had been prepared in complete medium. Each treatment was performed with three technical replicates.

After the cells adhered, 20 μL of MTT solution (5 g/L, Biosharp) was added to each well, followed by incubation at 37°C for 4 h [1]. The supernatant was then removed, and 150 μL of DMSO was added to each well. Then the plates were gently shaken for 10 min. Absorbance was measured at a wavelength of 490 nm.

Table S1. Effect of S50 on CTX-TNA2 cells viability

Concentration (mg/mL)	cells viability (%)
0	100
0.01	90.32±0.23
0.1	87.78±1.02
1	95.08±0.42

Reference:

1. Meng, L.S.; Xing, G.; Li, B.; Li, D.N.; Sung, X.Y.; Yan, T.C.; Li, L.; Cao, S.; Meng, X.J. Anthocyanins Extracted from <i>Aronia melanocarpa</i> Protect SH-SY5Y Cells against Amyloid-beta (1-42)-Induced Apoptosis by Regulating Ca²⁺ Homeostasis and Inhibiting Mitochondrial Dysfunction. *Journal of Agricultural and Food Chemistry* **2018**, *66*, 12967-12977, doi:10.1021/acs.jafc.8b05404.
