# Supplementary material for: The Effects of Semen Ziziphi Spinosae Extract on LPS-Induced Astrocyte Gene Expression and Metabolites
Source: Nutrients. 2025 Nov 7;17(22):3498. doi: 10.3390/nu17223498 (PMC12655257; doi:10.3390/nu17223498)
Supplement: Supplementary file 1 [file nutrients-17-03498-s001.zip › Supplementary File S2.pdf]

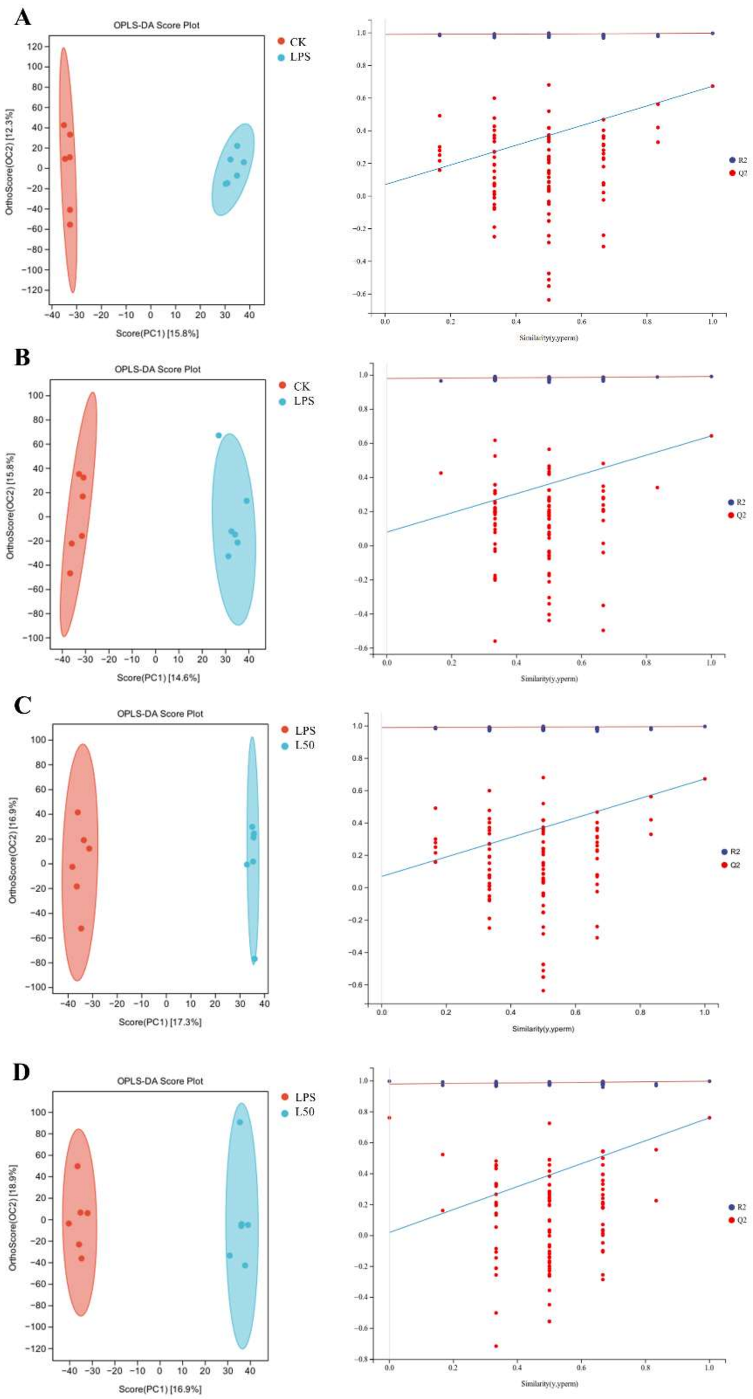

Figure S1. OPLS-DA score plots and permutation test results of different groups. Under positive ion mode (A and C); under negative ion mode (B and D).

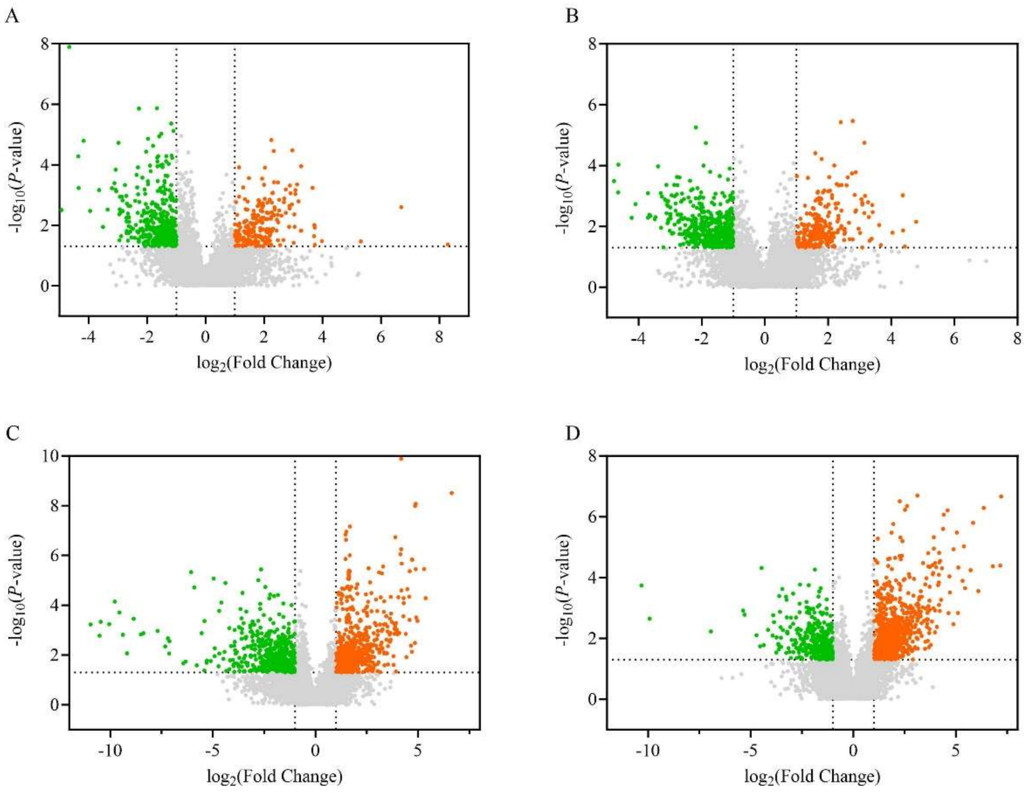

Figure S2. Volcano maps display the differential metabolites in LPS treatment group (LPS) compared with the control group under positive ion mode (A); the LPS group compared with the control group under negative ion mode (B); the LPS group compared with the LPS + 50% ethanol extract group (L50) under positive ion mode (C); the LPS group compared with L50 group under negative ion mode (D).
