# Supplementary material for: The Effects of Semen Ziziphi Spinosae Extract on LPS-Induced Astrocyte Gene Expression and Metabolites
Source: Nutrients. 2025 Nov 7;17(22):3498. doi: 10.3390/nu17223498 (PMC12655257; doi:10.3390/nu17223498)
Supplement: Supplementary file 1 [file nutrients-17-03498-s001.zip › Supplementary File S3.pdf]

Table S2. Primer information for the experimental process

Gene name	Gene ID	Primer F（5′-3′）	Primer R（5′-3′）
Cpt1a	ENSRNOG00000014254	ACAGACACCATCCAACATA	TAGAGCCAGACCTTGAAG
Foxo4	ENSRNOG00000033316	TCTCCTACTGAAGATTTC	ACTGATGATGTTATCCAT
Foxo3	ENSRNOG00000000299	CAGTGACTTGGACCTGGA	ATGAGTTCGCTACGGATGA
Stradb	ENSRNOG00000010728	GATTGTTTCTGTGCTTCA	AACCGAGTATTGATGGATA
Pfkm	ENSRNOG00000057988	ATCTCCAGGTTAATGTTG	AATCAGTAGTGTAGTTCTC
Ccnb1	ENSRNOG00000058539	GCAGTGAGTGATGTAGATG	GTTGTCTGAGGTAAGCATAG
Atg12	ENSRNOG00000000157	AGCAGTTGTTTATTTATGTG	AGTAATGTAGGACCAGTT
Ppp2ca	ENSRNOG00000005389	GGAGACTATGTGGACAGA	CCTCGGAGTATGGTGATA
Gabarapl1	ENSRNOG00000053362	GCCAGTTCTACTTCTTAATC	AGGGATAGTGTTGTTGAC
Atg101	ENSRNOG00000007756	AGGCAAGTTTCACTACAA	AAGTCACAGTCTACATCC
Lmna	ENSRNOG00000019638	TCCAGAAGAACATTTACA	ATTATCAATCTCCACCAG
Ctsf	ENSRNOG00000019708	AGACAGAGGATGACTATG	GAATCGTTGATGTAGACTT
gapdh	ENSRNOG00000018630	AAACCCATCACCATCTTC	TAGACTCCACGACATACT

Table S3. RNA sequencing statistics of clean sequencing data.

Sample	Raw reads	Clean reads	Clean (%)	Reads	Q20 (%)	Q30 (%)	GC (%)
CK1	51647746	51081766	98.90		98.72	96.15	48.50
CK2	44175768	43549328	98.58		98.44	95.35	48.53
CK3	40082710	39598296	98.79		98.58	95.74	48.44
LPS1	41319492	40806068	98.76		98.61	95.85	48.77
LPS2	41809772	41270038	98.71		98.55	95.69	48.54
LPS3	44013410	43456142	98.73		98.62	95.88	48.56
L501	37159904	36692582	98.74		98.57	95.75	48.60
L502	44151528	43632786	98.83		98.71	96.16	48.58
L503	44065166	43525530	98.78		98.63	95.90	48.47

Table S4. Statistics on the alignment of clean reads and reference genomes.

Sample	Total reads	Total mapped	Multiple mapped	Unique mapped
CK1	51081766	44100898 (86.33%)	1375519 (3.12%)	42725379 (96.88%)
CK2	43549328	37745344 (86.67%)	1193421 (3.16%)	36551923 (96.84%)
CK3	39598296	34566818 (87.29%)	1084290 (3.14%)	33482528 (96.86%)
LPS1	40806068	35312846 (86.54%)	1100990 (3.12%)	34211856 (96.88%)
LPS2	41270038	35763114 (86.66%)	1133734 (3.17%)	34629380 (96.83%)
LPS3	43456142	37737560 (86.84%)	1191991 (3.16%)	36545569 (96.84%)
L501	36692582	31838435 (86.77%)	1084678 (3.41%)	30753757 (96.59%)
L502	43632786	38030406 (87.16%)	1289037 (3.39%)	36741369 (96.61%)
L503	43525530	38081369 (87.49%)	1283487 (3.37%)	36797882 (96.63%)

Table S5. Overlapping Genes in Four Pathways Between Two Comparative Groups.

Pathway name	Gene ID	Gene name	CK vs. LPS Fold Change	CK vs. LPS Regulated	LPS vs. L50 Fold Change	LPS vs. L50 Regulated	Description
AMPK signaling pathway	ENSRNOG00000005389	Ppp2ca	1.1107	Up	0.7971	Down	protein phosphatase 2 catalytic subunit alpha [Source:RGD Symbol;Acc:3380]
	ENSRNOG00000003919	Rps6kb1	1.2292	Up	0.7826	Down	ribosomal protein S6 kinase B1 [Source:RGD Symbol;Acc:620683]
	ENSRNOG00000014254	Cpt1a	1.2033	Up	0.7246	Down	carnitine palmitoyltransferase 1A [Source:RGD Symbol;Acc:2396]
	ENSRNOG00000001309	Camkk2	1.2087	Up	0.5392	Down	calcium/calmodulin-dependent protein kinase kinase 2 [Source:RGD Symbol;Acc:620092]
	ENSRNOG00000000145	Pik3r3	1.2166	Up	0.5653	Down	phosphoinositide-3-kinase regulatory subunit 3 [Source:RGD Symbol;Acc:621042]
	ENSRNOG00000057988	Pfkm	0.7263	Down	1.3597	Up	phosphofructokinase, muscle [Source:RGD Symbol;Acc:68419]
	ENSRNOG00000000299	Foxo3	0.7377	Down	1.5152	Up	forkhead box O3 [Source:RGD Symbol;Acc:1309196]
	ENSRNOG0000001072	Stradb	0.7307	Down	1.6647	Up	STE20 related adaptor beta

	8						[Source:RGD Symbol;Acc:1559449]
	ENSRNOG00000022999	Ppp2r3a	0.7053	Down	1.4780	Up	protein phosphatase 2, regulatory subunit B'', alpha [Source:RGD Symbol;Acc:1306958]
	ENSRNOG00000037505	Ulk1	0.6947	Down	1.5556	Up	unc-51 like autophagy activating kinase 1 [Source:RGD Symbol;Acc:1589743]
	ENSRNOG00000029986	Insr	0.8057	Down	1.4284	Up	insulin receptor [Source:RGD Symbol;Acc:2917]
	ENSRNOG00000013397	Foxo1	0.6515	Down	1.4761	Up	forkhead box O1 [Source:RGD Symbol;Acc:620283]
	ENSRNOG00000019638	Lmna	1.3000	Up	0.6938	Down	lamin A/C [Source:RGD Symbol;Acc:620456]
	ENSRNOG00000023258	Nfkb1	1.4023	Up	0.6249	Down	nuclear factor kappa B subunit 1 [Source:RGD Symbol;Acc:70498]
	ENSRNOG00000021438	Tuba1c	1.2137	Up	0.6345	Down	tubulin, alpha 1C [Source:RGD Symbol;Acc:1307226]
Apoptosis	ENSRNOG00000025742	Lmnb2	1.2561	Up	0.7238	Down	lamin B2 [Source:RGD Symbol;Acc:1563803]
	ENSRNOG00000012331	Casp8	1.3358	Up	0.6228	Down	caspase 8 [Source:RGD Symbol;Acc:620945]
	ENSRNOG00000000145	Pik3r3	1.2166	Up	0.5653	Down	phosphoinositide-3-kinase regulatory subunit 3 [Source:RGD Symbol;Acc:621042]

Foxo signaling pathway	ENSRNOG00000013603	Dffa	1.2639	Up	0.6703	Down	DNA fragmentation factor subunit alpha [Source:RGD Symbol;Acc:620334]
	ENSRNOG00000019708	Ctsf	0.6913	Down	1.4063	Up	cathepsin F [Source:RGD Symbol;Acc:1308181]
	ENSRNOG00000058539	Ccnb1	1.4088	Up	0.6506	Down	cyclin B1 [Source:RGD Symbol;Acc:2291]
	ENSRNOG00000018815	Plk1	1.4168	Up	0.6599	Down	polo-like kinase 1 [Source:RGD Symbol;Acc:3352]
	ENSRNOG00000026109	Prmt1	1.2192	Up	0.8239	Down	protein arginine methyltransferase 1 [Source:RGD Symbol;Acc:62020]
	ENSRNOG00000000145	Pik3r3	1.2166	Up	0.5653	Down	phosphoinositide-3-kinase regulatory subunit 3 [Source:RGD Symbol;Acc:621042]
	ENSRNOG00000053362	Gabarapl1	0.6427	Down	1.9819	Up	GABA type A receptor associated protein like 1 [Source:RGD Symbol;Acc:1596143]
	ENSRNOG00000000157	Atg12	0.8277	Down	1.3396	Up	autophagy related 12 [Source:RGD Symbol;Acc:1306306]
	ENSRNOG00000033316	Foxo4	0.8108	Down	1.3206	Up	forkhead box O4 [Source:MGI Symbol;Acc:MGI:1891915]
	ENSRNOG00000029986	Insr	0.8057	Down	1.4284	Up	insulin receptor [Source:RGD Symbol;Acc:2917]
	ENSRNOG0000001339	Foxo1	0.6515	Down	1.4761	Up	forkhead box O1 [Source:RGD

	7						Symbol;Acc:620283]
Autophagy	ENSRNOG00000009867	Tgfb3	0.7795	Down	1.2524	Up	transforming growth factor, beta 3 [Source:RGD Symbol;Acc:3851]
	ENSRNOG00000005389	Ppp2ca	1.1107	Up	0.7971	Down	protein phosphatase 2 catalytic subunit alpha [Source:RGD Symbol;Acc:3380]
	ENSRNOG00000005165	Smcr8	1.3165	Up	0.7541	Down	SMCR8-C9orf72 complex subunit [Source:RGD Symbol;Acc:1564621]
	ENSRNOG00000003919	Rps6kb1	1.2292	Up	0.7826	Down	ribosomal protein S6 kinase B1 [Source:RGD Symbol;Acc:620683]
	ENSRNOG00000001309	Camkk2	1.2087	Up	0.5392+	Down	calcium/calmodulin-dependent protein kinase kinase 2 [Source:RGD Symbol;Acc:620092]
	ENSRNOG00000000145	Pik3r3	1.2166	Up	0.5653	Down	phosphoinositide-3-kinase regulatory subunit 3 [Source:RGD Symbol;Acc:621042]
	ENSRNOG00000053362	Gabarapl1	0.6427	Down	1.9819	Up	GABA type A receptor associated protein like 1 [Source:RGD Symbol;Acc:1596143]
	ENSRNOG00000007331	Rragd	0.4498	Down	2.6148	Up	Ras-related GTP binding D [Source:RGD Symbol;Acc:1310007]
	ENSRNOG0000003750	Ulk1	0.6947	Down	1.5556	Up	unc-51 like autophagy

5						activating kinase 1 [Source:RGD Symbol;Acc:1589743]
ENSRNOG00000000157	Atg12	0.8277	Down	1.3396	Up	autophagy related 12 [Source:RGD Symbol;Acc:1306306]
ENSRNOG00000037247	Rras	0.8012	Down	1.3422	Up	RAS related [Source:RGD Symbol;Acc:1311443]
ENSRNOG00000007756	Atg101	0.8155	Down	1.3266	Up	autophagy related 101 [Source:RGD Symbol;Acc:1359310]
ENSRNOG00000025443	Map1lc3a	0.8187	Down	1.2321	Up	microtubule-associated protein 1 light chain 3 alpha [Source:RGD Symbol;Acc:735183]

Table S6. The parameters of OPLS-DA analysis.

Multivariate analysis	Group	Positive ion model			Negative ion model		
		R^2X(cum)	R^2Y(cum)	Q^2(cum)	R^2X(cum)	R^2Y(cum)	Q^2(cum)
OPLS-DA	CK vs. LPS	0.281	0.997	0.673	0.305	0.992	0.644
	LPS vs. L50	0.342	0.998	0.762	0.358	0.995	0.763
